# Postoperative adjuvant radiation improves local control in surgically treated FIGO stage I-II small cell carcinoma of the cervix

**DOI:** 10.1186/s13014-019-1409-7

**Published:** 2019-11-13

**Authors:** Ting Shen, Yan-hui Jiang, Yao-yao Zou, Fang-fang Qiu, Xing-sheng Qiu, Kai-yun You

**Affiliations:** 1 Department of Radiation Oncology, Sun Yat-Sen Memorial Hospital, Sun Yat-Sen University, 107 Yanjiang Rd. West, Guangzhou, 510000 China; 2 Department of Rheumatology, Sun Yat-Sen Memorial Hospital, Sun Yat-Sen University, Guangzhou, China

**Keywords:** Small cell carcinoma of the cervix, Prognosis, Adjuvant radiation

## Abstract

**Objective:**

To determine the prognostic effect of adjuvant radiation and clinicopathological variables in surgically treated patients with small cell carcinoma of the cervix (SCCC).

**Methods:**

Clinical data of SCCC patients with International Federation of Gynaecology and Obstetrics (FIGO) stage I-II underwent radical surgery from May 2000 to August 2014 at Sun Yat-sen Memorial Hospital were retrospectively reviewed. Forty-three patients with SCCC were included to this study. Chi-square test or Fisher’s exact test, Student’s t test or Mann–Whitney U test, Kaplan–Meier method and multivariate analysis of Cox proportional hazards regression were used for statistical analysis. *P* < 0.05 was considered to be statistically significant.

**Results:**

Among 43 patients (median age, 49 years old) recruited, 25(58.1%) had stage I, 18(41.9%) had stage II disease. The 5-year overall survival (OS) rate was 39.54%, and the 5-year disease free survival (DFS) was 27.91%. Distant metastasis was the main cause of treatment failure (71.9%). Patients with adjuvant chemoradiation displayed lower rate of local recurrence than those with adjuvant chemotherapy (10.7% vs 60.0%, *P* < 0.0001). Multivariable analysis identified lymph node metastasis as a significant prognostic factor for both DFS and OS (*P* = 0.001, 0.004 respectively). Age was also an independent predictor of OS (*P* = 0.004). Adjuvant radiation appeared to significantly improve DFS (HR = 0.383, 95% CI, 0.185–0.791), but not OS.

**Conclusions:**

Adjuvant radiotherapy could improve the local control and prolong DFS in surgically treated SCCC. However, a large prospective clinical trial is needed to confirm this.

## Introduction

Small cell carcinoma of the cervix (SCCC) is a rare histological entity of cervical cancer with an incidence of less than 3% of all cervical cancers [1, 2]. Similar to those arising from the lung, rectum, and pancreas, though much rarer, small cell cervical carcinoma is highly aggressive and easy to present with nodal involvement and distant metastasis even with early-stage disease [3–5]. In a multi-center retrospective study of 188 patients with small cell cervical cancer, 5-year survival rate of International Federation of Gynaecology and Obstetrics (FIGO) stages I-IIA and IIB-IV were 36.8 and 8.9%, respectively [2]. Moreover, Viswanathan et al. reported that there were no survivors beyond 30 months in the patients with greater than IB1 disease [6]. In the past few decades, there was a significant trend for improved survival for both adenocarcinoma and squamous cell carcinoma of the cervix, but no detectable improvement for small cell carcinoma of the cervix [3].

Because of its low incidence and high mortality, most reported series have been small and from single institution [1, 7, 8]. Prospective clinical trial in SCCC is very scarce [7, 9, 10]. There were not clear treatment guidelines specific for SCCC. Reports and reviews in the literature provide some generalizations regarding treatment. The Society of Gynecologic Oncology (SGO) has recommended radical surgery for early-stage disease, and for patients with advanced-stage disease, chemoradiation or systemic chemotherapy was suggested [11]. Many researchers have proposed adjuvant chemotherapy due to the aggressive behavior of SCCC [1, 12]. At present, a growing number of studies have reported that adjuvant chemotherapy improve survival for patients with early stage small cell cervical carcinoma [1, 2, 13–15].

To our knowledge, there are currently few published studies which have compared the oncologic outcome between patients with or without receiving adjuvant radiotherapy in surgically treated early-stage disease. The significance and effectiveness of adjuvant radiotherapy are not yet clear. In fact, the Gynecologic Oncology Group (GOG) once sponsored a trial to study SCCC in Protocol 66 between 1982 and 1986, but failed to enroll enough patients for this trial [14]. For these reasons, reviews and researches based on large series of patients are of great importance. Hence we initiated this study to determine the prognostic factors and potential therapeutic modalities that may improve the outcomes in SCCC patients.

## Materials and methods

### Ethics statement

This research was approved by the Ethics Committee of Sun Yat-sen Memorial Hospital, and written informed consent was obtained from each patient included in the study.

### Patients and procedures

The data were extracted from a database that enrolled all the cervical cancer patients who underwent surgery at Sun Yat-sen Memorial Hospital between May 2000 and August 2014. The information on patient characteristics, pathologic diagnosis, treatment, and follow-up data was clearly recorded. As for the present study, the selection process and criteria were as follows (Additional file 1): (1) pathologically confirmed small cell carcinoma of the cervix or mixed; (2) received surgery followed by adjuvant therapy; (3) no sign of distant metastases during the treatment; and (4) no concurrent malignancy or prior history of radiation to the pelvis. Besides, all patients were staged according to the FIGO 2009 staging system. Patients with FIGO stage III-IV or incomplete medical records described above were excluded. All clinicopathological data were recorded, including age, FIGO stage, histological type, tumor size, lymphovascular invasion (LVSI), regional lymph node metastasis, adjuvant radiation and dose, Chemotherapy regimen and cycles.

### Chemotherapy

All the Patients were administered with systemic adjuvant chemotherapy. Chemotherapy were usually given every 3 weeks, whether concurrent with, after, or preceding radiation. There were two chemotherapy regimens as follows: etoposide (E) plus cisplatin or its analogs (P), paclitaxel and its analogs (T). And the median cycle of adjuvant chemotherapy were 5(4–6).

### External beam radiotherapy

Patients received a standard protocol of postoperative radiation with three-dimensional conformal RT (3D-CRT). The prescribed dose to the whole pelvis was 45–50 Gy, which was delivered in 1.8–2.0-Gy fractions once daily for 5 days per week. The clinical target volume (CTV) included the primary tumor bed, supra-vaginal portion, para-cervical tissue, common iliac lymph nodes, internal and external iliac lymph nodes, obturator lymph nodes and sacral lymph nodes. Roughly, the superior border of the CTV was the bottom of L4, and the inferior border was the lower margin of the obturator. The anterior border was the posterior margin of the bladder. When lateral fields were used, the posterior border encompassed S2.

### Follow-up evaluation

Patients were evaluated every 3 months for the first 2 years, every 6 months during the following 3 years, and annually thereafter. Complete blood cell counts, biochemical routines, NSE, and physical examinations were the routine evaluations during each visit. Vaginal cytology assessments were also performed for the detection of lower genital tract neoplasia. Chest radiography and computed tomography (CT) or magnetic resonance (MR) imaging of the abdomen and pelvis were conducted every 6 months, aimed to detect possible recurrent disease. If recurrent signs were suspected in a patient, biopsy was performed whenever possible. In our study, Local recurrence was defined as recurrence or progression within the pelvis. Systemic metastases was defined as recurrence outside the pelvis or tumor metastasizes to distant organs.

### Statistical analysis

All statistical analyses were performed using SPSS software, version 19.0. Categorical variables were analyzed using chi-square test or Fisher’s exact test. Continuous variables were analyzed using Student’s t test or Mann–Whitney U test. Kaplan–Meier method was used to compare disease free survival (DFS) and overal survival (OS) rates. Multivariate analysis of DFS and OS was analyzed using Cox proportional hazards regression, and the Cox proportional hazards model was performed using a forward conditional selection of variables. *P* < 0.05 was considered to be statistically significant.

## Results

### Clinical baseline characteristics

There were 43 patients enrolled in this study, including 25(58.1%) with stage I, and 18(41.9%) with stage II disease. The median age was 49 years old. Of them, there were 31 patients presented with pure pathology of small cell carcinoma, while the other 12 patients with mixed pathologic biology. There were 19 patients who were found with lymph node metastasis, and the other 24 patients were negative. Adjuvant radiation was administered in 28 patients and the other 15 patients received adjuvant chemotherapy alone. There were 23 patents presenting as large size tumors (tumor size≥4 cm) and the remaining 20 patients were with small size tumors (tumor size< 4 cm). LVSI was detected in 23 patients and the other 20 patients showed no LVSI (Table 1).

**Table 1:** Patient Demographics, Baseline Tumor Characteristics

Variables	Number	Percentage
Age, years		
≥ 49	20	46.5%
< 49	23	53.5%
FIGO stage		
IB1	11	25.6%
IB2	14	32.6%
IIA1	9	20.9%
IIA2	9	20.9%
Tumor biology		
pure	31	72.1%
mix	12	27.9%
Lymph node metastasis		
yes	19	44.2%
no	24	55.8%
Adjuvant radiation		
yes	28	65.1%
no	15	34.9%
Tumor size, cm		
≥ 4	23	53.5%
< 4	20	46.5%
LVSI		
yes	23	53.5%
no	20	46.5%
Chemotherapy regimen		
TP	30	69.8%
EP	13	30.2%

*Abbreviation*: *LVSI* Lymph-vascular space invasion, *EP* Etoposide (E) plus cisplatin or its analogs (P), *TP* Paclitaxel and its analogs (T) plus cisplatin or its analogs (P)

### Survival analysis for the whole group

**Fig. 1:**
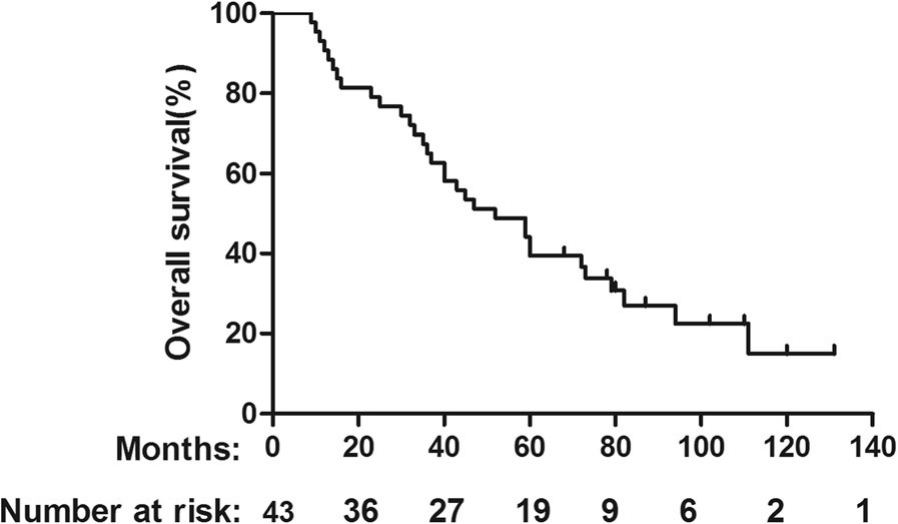
OS for the whole group stratified by adjuvant radiation

**Fig. 2:**
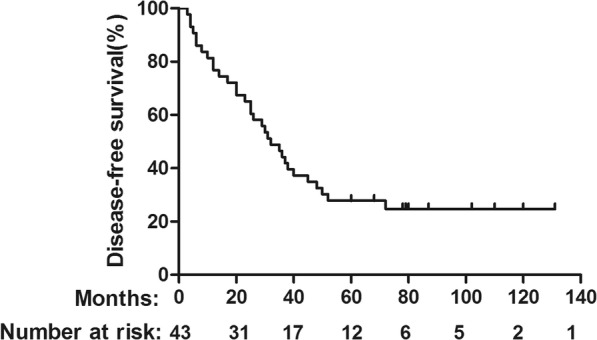
DFS for the whole group stratified by adjuvant radiation

Among all patients, the median follow up was 52 months, 32 patients died during follow-up. The 5-year OS rates were 39.54% (Fig. 1). Recurrence was found in 32 patients and the 5-year DFS was 27.91% (Fig. 2). Among them, 9 patients developed local recurrence and 20 patients presented with distant metastasis. There were 3 patients who developed both local and distant failure. For patients received adjuvant chemoradiation, there were 3 patients developed local recurrence and 15 patients presented with distant metastasis. And for patients received adjuvant chemotherapy alone, there were 6 patients developed local recurrence and 5 patients presented with distant metastasis, 3 patients who developed both local and distant failure. In details, the sites of local recurrence were primary tumor region or vaginal stump (total 9 cases) and drainage area of internal iliac lymph (3 cases). Distant metastasis was the main cause of treatment failure (71.9%). Among the patients with distant failure, 9 patients developed only liver metastasis and 8 patients developed only lung metastasis. There were 3 patients who were with both liver and lung metastasis. Bone metastasis was found in 2 patients and 1 patients presented metastasis to paraaortic lymph nodes.

### Univariate and multivariable analysis of DFS and OS for the whole group

By univariate analysis, we found that patients with positive lymph node metastasis displayed poorer DFS than those without lymph node metastasis and patients received adjuvant chemoradiation showed longer DFS than those who received adjuvant chemotherapy alone. Besides, young patients tended to achieve a better OS than the elderly patients. Furthermore, patients without lymph node metastasis also showed improved OS than those with positive lymph node metastasis (Table 2). Through further multivariable analysis we found that both adjuvant radiation and lymph node metastasis were independent predictors of DFS. Additionally, lymph node metastasis was also significantly associated with impaired OS and younger age significantly predicted more favorable OS (Table 3).

**Table 2:** Univariate analysis of DFS and OS for The Whole Group

Variable	Number of patients	5-year DFS	*P* value	5-year OS	*P* value
Age, year			0.063		0.044
≥ 49	20	15.0%		25.0%	
< 49	23	39.1%		50.6%	
FIGO Stage			0.352		0.414
I	25	28.0%		34.3%	
II	18	27.8%		44.4%	
Tumor biology			0.398		0.554
pure	31	25.8%		38.7%	
mixed	12	33.3%		38.9%	
Lymph node metastasis			0.003		0.035
yes	19	10.5%		31.6%	
no	24	41.7%		44.9%	
Adjuvant radiation			0.038		0.097
yes	28	35.7%		42.9%	
no	15	13.3%		31.1%	
Tumor size, cm			0.342		0.116
≥ 4	23	26.1%		30.4%	
< 4	20	30.0%		48.7%	
LVSI			0.388		0.209
yes	23	26.1%		33.8%	
no	20	30.0%		45.0%	

*Abbreviation*: *DFS* Disease-free survival, *OS* Overall survival, *LVSI* Lymph-vascular space invasion

**Table 3:** Multivariate analysis of DFS and OS for The Whole Group

Variable	DFS		OS	
	HR(95%CI)	*P* value	HR(95%CI)	*P* value
Lymph node metastasis no vs yes	0.282 (0.136–0.588)	0.001	0.326 (0.153–0.695)	0.004
Adjuvant radiation yes vs no	0.383 (0.185–0.791)	0.009	_	
Age, year < 49 vs ≥49	_		0.329 (0.154–0.703)	0.004

*Abbreviations*: *DFS* Disease-free survival, *OS* Overall survival, *CI* Confidence interval, *HR* Hazard ratio

### Subgroup analysis based on clinical factor of adjuvant radiation

**Fig. 3:**
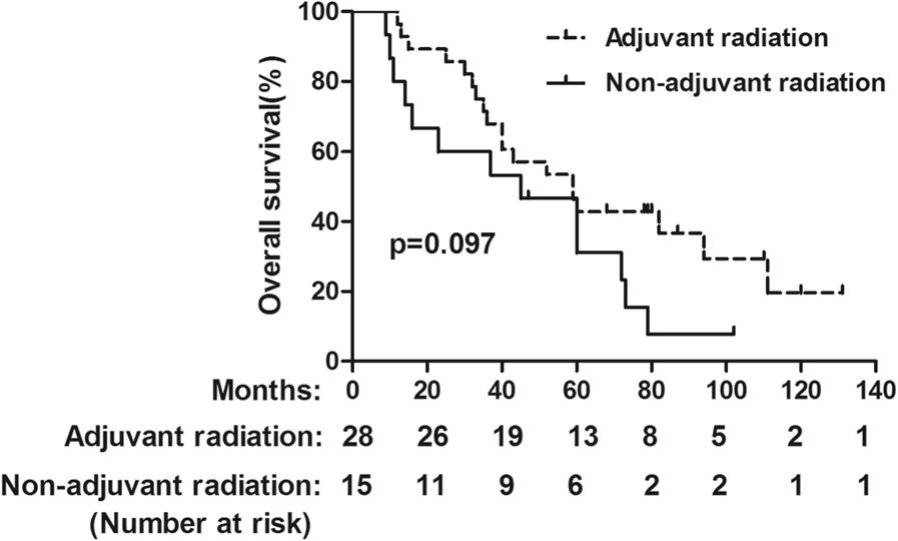
OS for the whole group

**Fig. 4:**
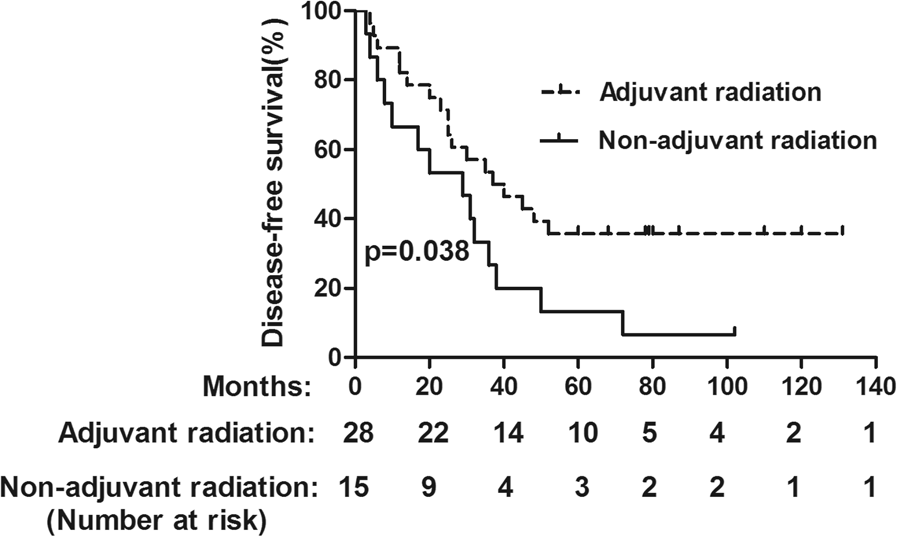
DFS for the whole group

As we have showed that the factor of adjuvant radiation was independent predictor of DFS, further subgroup analysis was done on the basis of balanced baseline characteristics between patients who did and did not receive adjuvant radiotherapy [Table 4]. The results showed that 5-year OS in the adjuvant chemoradiation group and adjuvant chemotherapy groups were 42.9 and 35.7%, respectively (Fig. 3). No significant differences were found in OS rate between the two groups (*p* = 0.097). However, the 5-year DFS in adjuvant chemo-radiation group was significantly higher than those in the adjuvant chemotherapy group (35.7% vs 13.3%, *p* = 0.038) (Fig. 4). The recurrence pattern was further analyzed with results showing that the patients in the adjuvant chemoradiation group displayed a lower rate of local recurrence than those in the adjuvant chemotherapy group (*p* < 0.001). Nevertheless, the rate of distant metastasis was similar between the two groups (*p* = 0.772) (Table 5).

**Table 4:** Comparison of baseline characteristics between patients who did and did not receive adjuvant radiotherapy

Variable	Adjuvant radiation (*n* = 28)	Non-adjuvant radiation (*n* = 15)	*P value*
Age, years			0.988
≥ 49	13	7	
< 49	15	8	
Tumor size, cm			0.512
≥ 4	16	7	
< 4	12	8	
FIGO stage			0.640
I (IB1 and IB2)	17	8	
II (IIA1 and IIA2)	11	7	
Lymph node metastasis			0.126
yes	10	9	
no	18	6	
LVSI			0.056
yes	12	11	
no	16	4	
Chemotherapy regimen			0.745
EP	9	4	
TP	19	11	
Overall follow up, months Median (range)	59 (12–131)	45 (9–102)	0.120

*Abbreviation*: *LVSI* Lymph-vascular space invasion, *EP* Etoposide (E) plus cisplatin or its analogs (P), *TP* Paclitaxel and its analogs (T) plus cisplatin or its analogs (P)

**Table 5:** Comparison of Recurrence Pattern between Patients Who Did and Did not Receive Adjuvant Radiation

Group	Adjuvant radiation	Non-adjuvant radiation	*P* value
	5-year	5-year	
LR	3 (10.7%)	9 (60.0%)	< 0.001^a^
SM	15 (53.6%)	8 (53.3%)	0.772^a^

Values are presented as number (%)

*Abbreviations*: *LR* Local recurrence, *SM* Systemic metastases

^a^: calculated by Kaplan–Meier method

## Discussion

Radical hysterectomy is commonly recommended in the primary treatment of early-stage SCCC patients. Likewise, another study based on 188 patients showed that a sharp difference in the 5-year OS rate between I-IIA patients who underwent radical hysterectomy and those who did not [2]. Radical hysterectomy remains an important component of the standard treatment in early stage small cell cervical cancer [11, 14–16]. Considering the aggressive nature of SCCC [5, 17], multimodality treatment should be considered. A growing number of studies favored radical surgery followed by adjuvant chemotherapy as the optimal treatment choice for stagesIand II SCCC patients [8, 15, 16]. Patients in our report all received adjuvant chemotherapy after radical surgery. Our study observed a 5-year survival rate of 39.54%, consistent with previous reports that, even for patients with early stage disease, the overall survival ranges from 30 to 60% [3, 7, 13–15]. However, the efficacy of adjuvant radiation for patients with early stage disease have not yet been clarified clearly [8, 15, 16]. We therefore carried out this retrospective study to figure out the efficacy of adjuvant radiation and identify the clinicopathological prognostic factors of survival, aimed to determine a better treatment strategy for patients with early stage SCCC.

Large tumor size, lymph node metastases, smoking, advanced FIGO stage, deep stromal invasion, pure histology type and without chemotherapy have been indicated as possible poor prognostic factors by previous literatures [3, 6, 7, 12–14, 17]. Consistent with other studies [3, 17], our study found that lymph node metastasis was an independent prognostic factor for OS and DFS. Patients with lymph node metastasis had poor prognosis. The elders over 49 years also showed poor survival. Other variables, including pure histologic type, large tumor size and stage were not statistically significant, most likely due to the sample size in our study was not big enough to obtain statistical significance.

Adjuvant radiotherapy was commonly recommended for postoperative cervical cancer patients with risk factors according to surgical specimens. Considering the aggressive nature of SCCC, additional radiation was often recommended, though the survival advantage of adjuvant radiotherapy was rarely reported. Our study elucidated that postoperative adjuvant chemoradiation significantly improved DFS than those who received adjuvant chemotherapy alone, but the difference in OS was not statistically significant. Lee et al. also hold that adjuvant chemoradiation did not improve OS compared with adjuvant chemotherapy alone [15], which was consistent with our finding. But Lee et al. did not compare DFS and the recurrence mode. Pei et al. made a comparison between patients with 5 cycles of EP (etoposide(E), cisplatin and its analogs (P)) chemotherapy or more plus radiation, and those with 5 cycles of EP or more but without radiation, the former did not improve recurrence-free survival [8]. The most possible reason was that the effects of radiation were antagonized by aggressive chemotherapy. In our study, further comparison of recurrence patterns showed that distant metastasis was the main cause of treatment failure (71.9%), and adjuvant radiotherapy significantly reduced local recurrence rate, but did not reduce distant metastasis. The lack of improvement in overall survival was likely due to the inability to prevent distant metastases.

## Conclusion

In conclusion, the present study indicated that lymph node status and age, which are independent prognostic factors, could act as additional prognostic survival factors. Moreover, our results indicated adjuvant chemoradiation significantly prolonged DFS and improved the local control than adjuvant chemotherapy alone. To our knowledge, this is the first report to confirm the benefit from adjuvant radiation. The results of the present study indicated primary radical surgery followed by adjuvant chemoradiation as the optimal treatment choice for patients with stageI-IISCCC disease. Because this was a single-institutional retrospective study, inherent biases were inevitable. Besides, the number of patients in our study was limited. Therefore, the finding should be validated by larger samples. Despite limitations of a retrospective study, our study provides an important basis for designing future prospective studies.

## Supplementary information

**Additional file 1: Figure S1.** Patients’ inclusion/exclusion process.

## Data Availability

The data supporting our findings can be found in the database in Sun Yat-sen Memorial Hospital.
