# Supplementary figures and images for: Postoperative adjuvant radiation improves local control in surgically treated FIGO stage I-II small cell carcinoma of the cervix
Source: Radiat Oncol. 2019 Nov 13;14:203. doi: 10.1186/s13014-019-1409-7 (PMC6854720; doi:10.1186/s13014-019-1409-7)

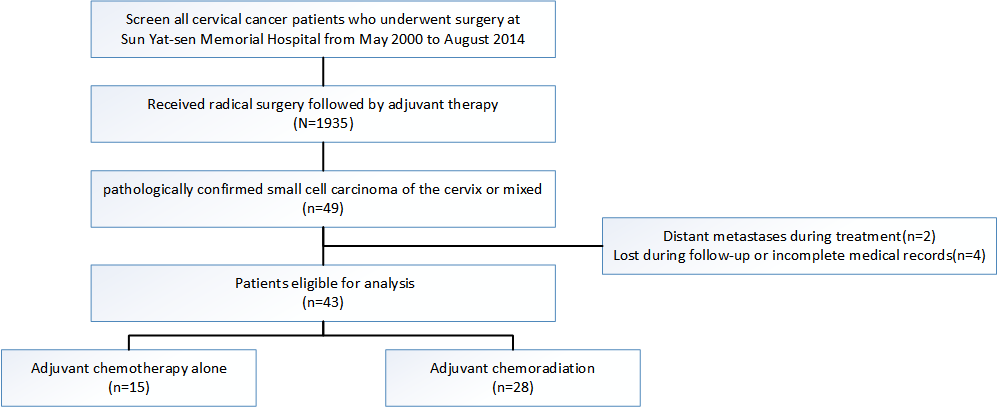

Supplement: Supplementary file 1 — Additional file 1: Figure S1. Patients’ inclusion/exclusion process. [file 13014_2019_1409_MOESM1_ESM.tif]
